# The Ability of the Nitric Oxide Synthases Inhibitor T1023 to Selectively Protect the Non-Malignant Tissues

**DOI:** 10.3390/ijms22179340

**Published:** 2021-08-28

**Authors:** Marina Filimonova, Alina Saburova, Victoria Makarchuk, Ljudmila Shevchenko, Valentina Surinova, Vadim Yuzhakov, Nina Yakovleva, Larisa Sevankaeva, Vyacheslav Saburov, Sergey Koryakin, Petr Shegay, Andrey Kaprin, Sergey Ivanov, Alexander Filimonov

**Affiliations:** 1 A. Tsyb Medical Radiological Research Center—Branch of the National Medical Research Radiological Center of the Ministry of Health of the Russian Federation, 249036 Obninsk, Russia; alinasamsonova.515@gmail.com (A.S.); vikymakarchuk@mail.ru (V.M.); schew.ludmila@yandex.ru (L.S.); val_suriniva@mail.ru (V.S.); yuzh_vad@mail.ru (V.Y.); yakovleva.40@mail.ru (N.Y.); larisa.sevankaeva@mail.ru (L.S.); vosaburov@gmail.com (V.S.); korsernic@mail.ru (S.K.); oncourolog@gmail.com (S.I.); filimonov_alex@mail.ru (A.F.); 2 National Medical Research Radiological Center of the Ministry of Health of the Russian Federation, 249036 Obninsk, Russia; dr.shegai@mail.ru (P.S.); kaprin@mail.ru (A.K.)

**Keywords:** nitric oxide synthase inhibitor, radiotherapy, radiation-induced tissue toxicity, selective radiation protection

## Abstract

Previously, we showed that a nitric oxide synthase (NOS) inhibitor, compound T1023, induces transient hypoxia and prevents acute radiation syndrome (ARS) in mice. Significant efficacy (according to various tests, dose modifying factor (DMF)—1.6–1.9 against H-ARS/G-ARS) and safety in radioprotective doses (1/5–1/4 LD10) became the reason for testing its ability to prevent complications of tumor radiation therapy (RT). Research methods included studying T1023 effects on skin acute radiation reactions (RSR) in rats and mice without tumors and in tumor-bearing animals. The effects were evaluated using clinical, morphological and histological techniques as well as RTOG classification. T1023 administration prior to irradiation significantly limited the severity of acute RSR. This was due to a decrease in radiation alteration of the skin and underlying tissues, and the preservation of the functional activity of cell populations that are critical in the pathogenesis of radiation burn. The DMF values for T1023 for skin protection were 1.4–1.7. Moreover, its radioprotective effect was fully selective to normal tissues in RT models of solid tumors—T1023 reduced the severity of acute RSR and did not modify the antitumor effects of γ-radiation. The results indicate that T1023 can selectively protect the non-malignant tissues against γ-radiation due to hypoxic mechanism of action and potentiate opportunities of NOS inhibitors in RT complications prevention.

## 1. Introduction

The frequency of cancer incidence is steadily increasing in almost all countries of the world. Over 3 million patients are currently diagnosed with cancer in the Russian Federation [1]. About 50–70% of patients receive different types of radiation therapy (RT) that remains one of the most effective tools in cancer therapy. The ongoing efforts towards designing new radiation treatment techniques are aimed to improve the quality of life of cancer patients and to minimize the toxicity of radiotherapy. Dose fractionation and conformal radiation techniques along with molecular targeted therapy have improved the preservation of normal cells/tissues during radiation treatment [2]. Despite such efforts, patients in 10–15% of cases (and in some local tumors, up to 40%) are faced with the complications arising from radiation damage to normal tissues [3,4,5]. Such complications are able to limit the possibility of cancer therapy in full. Moreover, some pathologies, especially late radiation injuries, which are based on the processes of fibrogenesis, are difficult to cure with conservative therapy [6,7]. In some cases, they can become threatening, capable of leading to the failure of internal organs and death [8,9]. It is obvious that minimizing such RT risks requires versatile efforts aimed at both further improving medical radiological techniques, methods of planning radiation exposure and, on the other hand, developing new approaches to pharmacological prevention and treatment of radiation injuries [10,11,12].

Earlier, we showed that some S-alkyl-N-acyl-substituted isothioureas are effective competitive inhibitors of nitric oxide synthases (NOS) capable of causing a pronounced vasoconstrictor, vasopressor effect [13,14]. Such vasotropic activity allows these compounds induce reflex changes in hemodynamics, leading to transient hypoxia, and exerts a preventive radioprotective effect [15,16,17]. In particular, we have shown that the NOS inhibitor 1-isobutanoyl-2-isopropylisothiourea hydrobromide (compound T1023) is highly effective (dose modifying factor (DMF)—1.6–1.9) in the prevention of H-ARS and G-ARS in mice [18]. The data on the T1023 significant radioprotective activity and the relative safety of its effective dose (1/5–1/4 LD10) are the reason for studying its ability to protect normal somatic tissues in models of acute radiation damage to the skin, as well as its radiation modifying effects in RT models.

## 2. Results

### 2.1. T1023 Limits the Severity of Acute Radiation Damage to Normal Tissues

#### 2.1.1. Clinical and Morphological Study in Rats

In the first experiment, a clinical and morphological effect of T1023 (75 mg/kg, single i.p., 30 min before local gamma irradiation (LI)) on the development of skin acute radiation reactions (RSR) on the right hind limb of Wistar rats was studied. The animals (10–11 rats per group) were observed for 25 days after γ-ray LI at doses of 32 or 36 Gy.

**Figure 1:**
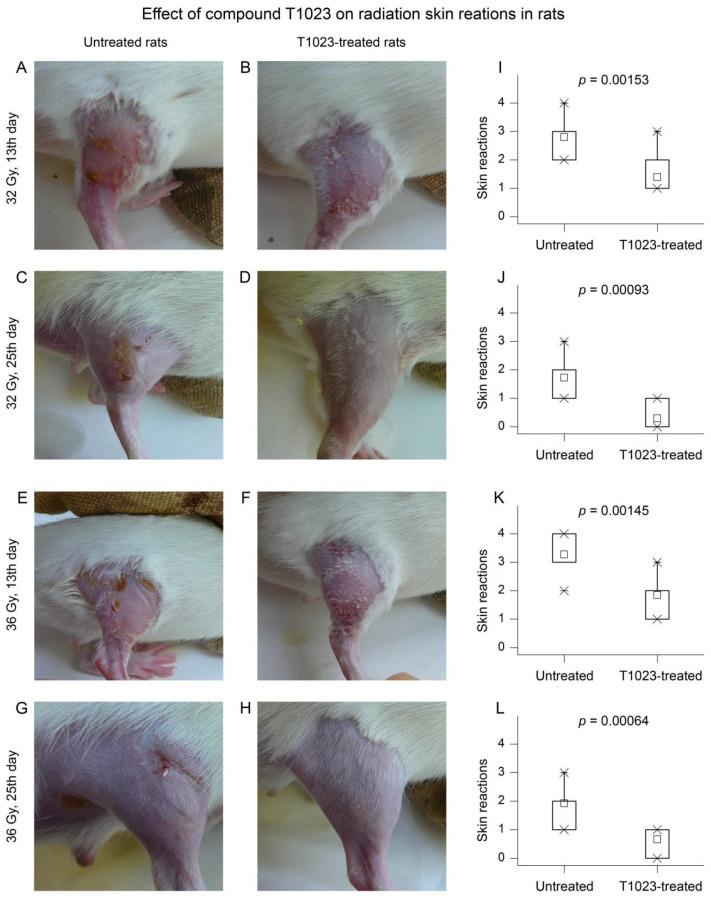
T1023 (75 mg/kg, single, i.p., 30 min before LI) influence on the RSR development in rats after γ-ray LI at doses of 32 and 36 Gy. (**A**–**H**) RSR typical macroscopic picture in control and T1023-treated Wistar rats at various time after γ-ray LI. (**A**) Control rat on 13th day after LI at 32 Gy—swelling of the skin, confluent EE, islet EE on the inner surface. (**B**) T1023-treated rat on 13th day after LI at 32 Gy—erythema, DD, moderate erythema on the inner surface. (**C**) Control rat on 25th day after LI at 32 Gy—incomplete epithelization after confluent EE, full epilation. (**D**) T1023-treated rat on 25th day after LI at 32 Gy—manifestation of RSR is absent, restoration of previously removed coat. (**E**) Control rat on 13th day after LI at 36 Gy—swelling of the skin, confluent EE, local ulceration, EE on the inner surface. (**F**) T1023-treated rat on 13th day after LI at 36 Gy—islet EE, DD on the inner surface. (**G**) control rat on 25th day after LI at 36 Gy—marginal epithelization of the ulcer with the formation of fibrosis tissue, point bleeding, full epilation. (**H**) T1023-treated rat on 25th day after LI at 36 Gy—unchanged on the external surface, partial epilation on the inner surface. (**I**–**L**) The distributions of RSR degree on RTOG scale in control and T1023-treated rats at various time after γ-ray LI; *p*—statistical level differences with control (*n* = 10–11 per group).

The macroscopic picture of the RSR initial development in control and T1023-treated rats at dose 32 Gy did not differ. Radiation reaction in these animals manifested 3–5 days after LI in the form of moderate erythema and signs of dry desquamation (DD), or loss of superficial layers of epidermis. From day 7 after LI, RSR manifestations in control rats increased rapidly. The edema increased and the epidermitis acquired an exudative character. By 10–13 days after LI in control rats, the severity of acute RSR corresponded to grades 2–3 according to the RTOG scale. On the lateral and inner surface of the irradiated limb, exudative, more often confluent epidermitis was observed, which was sometimes complicated by small ulcerations and petechiae (Figure 1A). The subsequent recovery processes in these animals lasted quite a long time. In some of the control rats, which had widespread confluent exudative epidermitis (EE), epithelization of the most damaged parts of the thigh remained incomplete until the end of the observation (Figure 1C). In T1023-treated rats, by days 10–13 after LI at 32 Gy, an increase in morphological manifestations of RSR was observed in a small part of animals. It was manifested only by increased erythema and the development of islet EE (Figure 1B). During this period, the severity of acute RSR in T1023-treated rats corresponded to grade 1–2 on the RTOG scale. Subsequently, the manifestations of RSR in these rats quickly regressed. By the 25th day of the experiment, 70% of these animals had no changes in the skin and its appendages on the irradiated limb (Figure 1D).

A similar effect of T1023 on the RSR development was manifested in Wistar rats after LI at 36 Gy. In this case, by days 10–13 after LI in control rats, the severity of acute RSR corresponded to 3–4 degrees according to the RTOG scale. On the background of pronounced skin edema, a widespread confluent EE developed in almost all animals. In 40% of cases, it was accompanied by bleeding and the formation of ulcerative defects (Figure 1E). Subsequently, such defects were restored due to edge epithelialization, including the formation of fibrous tissue (Figure 1G). By the 25th day of the experiment, the regeneration of skin lesions remained incomplete in most rats of this group.

By days 10–13 after LI at 36 Gy, the severity of acute RSR in most of the T1023-treated rats corresponded to grade 2 according to the RTOG scale-moderate skin edema and islet EE developed (Figure 1F). However, the confluent form was observed only in 20% of these animals, and necrotic ulcerative complications were not registered. By the 25th day of the experiment, in the majority of T1023-treated rats, minimal manifestations of RSR persisted—slight desquamation of the epidermis (Figure 1H). Epithelialization after confluent EE remained incomplete in only one animal.

T1023, in this experiment, effectively reduced the morphological severity of RSR. It is confirmed by the high statistical significance of differences in RSR degree according to the RTOG scale between the control and T1023-treated rats (Figure 1I–L).

In general, at both doses of LI, the clinical picture of RSR in control rats reflected a significant, often complete, radiation damage to the epidermis and partial destruction of the deep layers of the dermis and epithelial appendages of the skin [3,19]. A decrease in the RSR degree and a relatively rapid regeneration of damaged tissues in T1023-treated rats, in our opinion, reflected the radioprotective effect of T1023 on normal somatic tissues. As a result, radiation alteration of the skin and underlying tissues was limited, and the functional viability of the epidermis and dermis was maintained at a level sufficient for effective physiological regeneration.

#### 2.1.2. Histological Study in Rats

Similar conclusions were also obtained from the results of histological studies of the tissues of the thigh of the irradiated hind limb in control and T1023-treated Wistar rats (75 mg/kg, single i.p., 30 min before LI) on the 15th day after γ-ray LI at dose 40 Gy (three rats per group).

**Figure 2:**
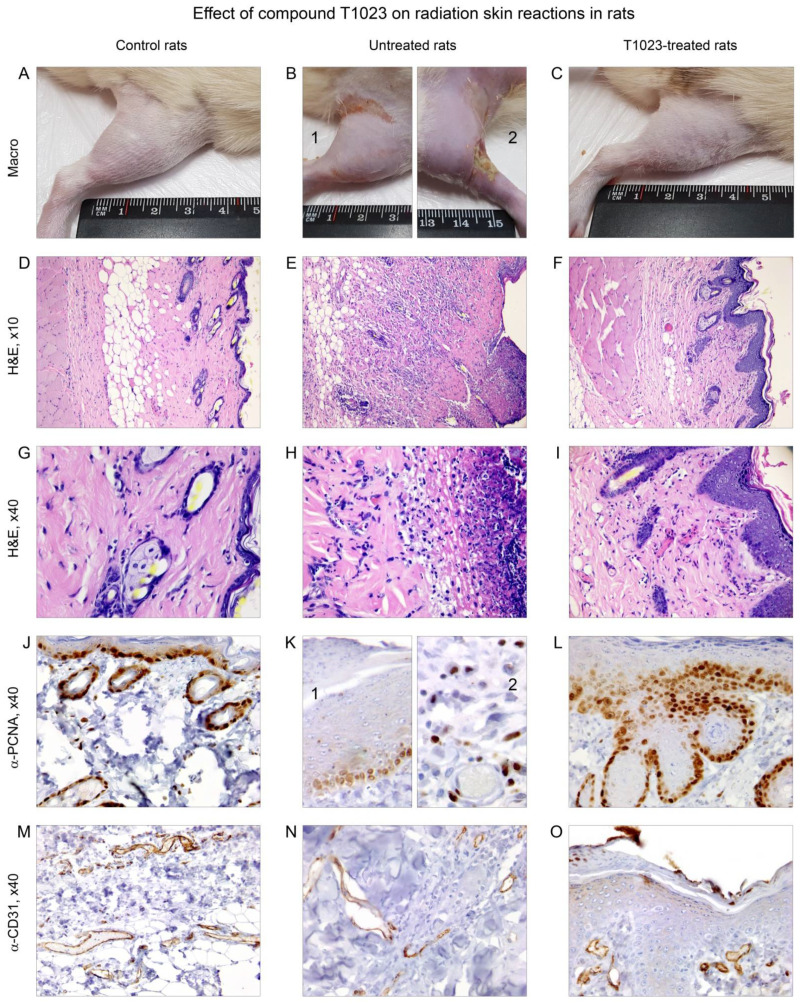
T1023 (75 mg/kg, single, i.p., 30 min before LI) influence on the morphology of rat hip tissues on 15th day after LI at 40 Gy. (**A**–**C**) Macroscopic pictures. (**A**) Intact rat—no features. (**B**) Untreated rats: 1—confluent EE (30 × 5 mm), spreading to the inner surface; 2—edema, EE, radiation ulcer (9 × 3 × 5 mm) with purulent contents. (**C**) T1023-treated rat—moderate hyperemia, DD. (**D**–**F**) General histological picture of the tissues of the thigh (H&E; ×35). (**D**) Intact rat—the histological structure of the skin and underlying tissues corresponds to the age norm (see text). (**E**) Untreated rat—lack of epidermis, the formation of thin scabs consisting of necrotic masses infiltrated by neutrophils, the development of small epithelial regenerates; in the compacted dermis, replacement of the remnants of destructed epithelial appendages of the skin with connective tissue is observed. (**F**) T1023-treated rat—the skin is almost completely epithelialized; the epidermis is thickened; the dermis is moderately edematous with preserved hair follicles and sebaceous glands in it; subcutaneous tissue and muscle fibers are unchanged. (**G**–**I**) Fragments of d-f, respectively (H&E, ×140). (**G**) Intact rat—interweaving of collagen fibers, fibrocytes and hair with adjacent sebaceous glands in the reticular layer of the dermis. (**H**) untreated rat—severe edema and infiltration of lymphocytes and neutrophils in the dermis. (**I**) T1023-treated rat—strengthening of the vasculature and overgrowth of loose connective tissue in the dermis. (**J**–**L**) Proliferating cells (α-PCNA, ×140). (**J**) intact rat—active cell proliferation in the basal layer of the epidermis, epithelium of hair follicles, endothelium of capillaries and fibroblasts. (**K**) Signs of regeneration in untreated rats: 1—moderate proliferation of cells of superficial epithelial regenerates; 2—cell proliferation of connective tissue and endothelium in the deep layers of the dermis and subcutaneous adipose tissue. (**L**) T1023-treated rat—high proliferative activity of cells in the basal layer of the epidermis and epithelial cells of the outer hair sheaths. (**M**–**O**) Vasculature (α-CD31, ×140). (**M**) Intact rat—the vascular network of the dermis and subcutaneous tissue are of usual structure. (**N**) Untreated rat—dilated full-blooded vessels of the deep layers of the dermis. (**O**) T1023-treated rat—newly formed vessels of the dermis.

For a comparative assessment, the histological structure of the skin and underlying tissues of the thigh of an intact rat was also studied. On visual examination, the skin on all surfaces of the thigh of an intact animal had no abnormalities (Figure 2A). The histological structure of the thigh tissue corresponded to age norms (Figure 2D). The epidermis was not damaged, thin, consisting of two layers of cells. The stratum corneum of the epidermis was formed by thin scales. The dermis was represented by a weakly expressed papillary layer, consisting of a loose fibrous connective tissue with single fibroblasts, and a reticular layer formed by a dense unformed connective tissue, in which the weaves of collagen fibers, fibrocytes and hair with adjacent sebaceous glands were located (Figure 2G). When staining for PCNA, active proliferation of cells of the basal layer of the epidermis, epithelium of hair follicles, endothelium of capillaries and fibroblasts were detected (Figure 2J). The vascular network of the dermis and subcutaneous tissue was of a normal structure (Figure 2M).

T1023-untreated rats on day 15 after γ-ray LI at dose 40 Gy external examination: RSR of the irradiated limb corresponded to grade 3–4 according to the RTOG scale. The skin of the thigh was swollen, hyperemic, with a bluish tinge. All rats had local erythematous spots and EE; in some rats, it was complicated by radiation ulcers (Figure 2B). Microscopic examination found that the epidermis was absent in the area of the most pronounced radiation damage. In zones of skin ulceration and tissue destruction, a small thickness of the scab was revealed, consisting of necrotic masses infiltrated by neutrophils, and not separated from the underlying tissues. The formation of small epithelial regenerates was also noted (Figure 2E). In the compacted dermis, the remnants of destructed epithelial appendages of the skin were visualized with their replacement by connective tissue, significant edema and infiltration with lymphocytes and neutrophils were noted (Figure 2H). Signs of the development of reparative regeneration in these animals were manifested by moderate cell proliferation of superficial epithelial regenerates (Figure 2(K1)), as well as vascular congestion (Figure 2N) and proliferation of connective tissue and endothelial cells (Figure 2(K2)) in the deep layers of the dermis and subcutaneous adipose tissue.

In T1023-treated rats on day 15 after γ-ray LI at dose 40 Gy external examination, the RSR on the irradiated limb corresponded to a grade 1–2 RTOG scale. No pronounced lesions on the skin of the thigh were observed; moderate hyperemia, signs of DD and single petechiae less than 1 mm in size were noted (Figure 2C). Microscopic examination found that the skin of the irradiated thigh in all T1023-treated rats looked almost completely epithelialized (Figure 2F). Moreover, the epidermis was thickened due to the proliferation of cells in the basal layer and the proliferation of a layer of spiny cells. The dermis was moderately edematous, and preserved hair and sebaceous glands were visualized. The subcutaneous adipose tissue and underlying muscle fibers looked unchanged. Connective tissue growth in the papillary and reticular dermis layers with newly formed vessels (Figure 2I,O) and the high proliferative activity of the epithelial cells of the outer layer hair follicles were noted (Figure 2L).

Comparison of the irradiated hip tissues in the control and T1023-treated rats testified that the use of T1023 at a dose effective for ARS prevention was accompanied by a significant decrease in the level of radiation-induced injuries, relatively rapid epithelialization of the radiation damage zone and normalization of the histological structure of the underlying tissues. These data indicate that the protective effect of T1023 was realized by limiting the damage and maintaining the functional activity of cells in the basal layer of the epidermis, epithelium of hair follicles, vascular endothelium and fibroblasts, i.e., cell populations that are most critical in the development of this radiation pathology [3,19].

#### 2.1.3. Clinical and Morphological Study in Mice

**Figure 3:**
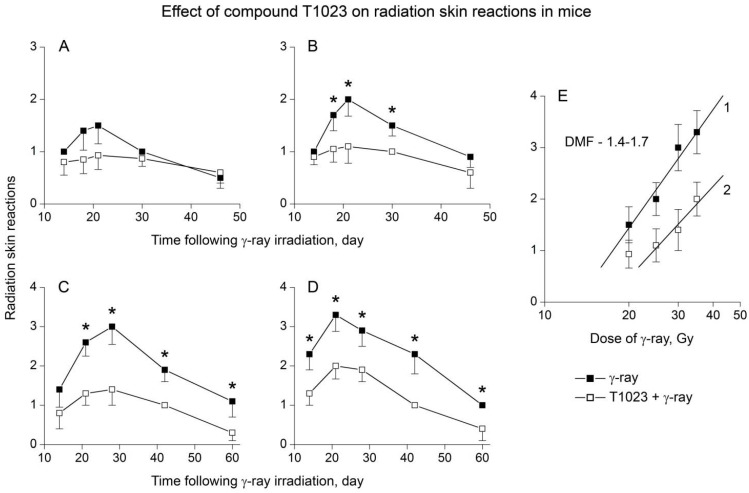
T1023 (75 mg/kg, single, i.p., 30 min before LI) influence on RSR development in mice after γ-ray LI. (**A**–**D**) RSR dynamics in control and T1023-treated mice after LI at doses of 20 (**A**), 25 (**B**), 30 (**C**) and 35 (**D**) Gy. Assessment of the RSR degree was carried out according to the RTOG scale. Graphical deviations correspond to SD (*n* = 14–15 per point). * Significantly different RSR in ±T1023 mice ((**B**): *p* = 0.02891, *p* = 0.00430, *p* = 0.01596; (**C**): *p* = 0.00177, *p* = 0.00019, *p* = 0.00648, *p* = 0.01875; (**D**): *p* = 0.00914, *p* = 0.00332, *p* = 0.00549, *p* = 0.00242, *p* = 0.02411, respectively); (**E**) the dependence of the maximal RSR degree in control and T1023-treated mice on the dose of γ-ray LI. Inclined lines 1, 2—corresponding log-linear regressions (*p* = 0.00972, *p* = 0.00523, respectively) for a range of moderate toxic effects (RSR—1.0–3.5). DMF—ratio of equal toxic doses values ± T1023, calculated by these regressions.

For detailed assessment of T1023 ability to protect normal somatic tissues, a series of experiments was carried out. The effect of T1023 (75 mg/kg, single i.p., 30 min before LI) on the dynamics of the development of acute RSR after γ-ray LI at doses of 20–35 Gy in white outbred mice was studied (14–15 mice per group). Results showed that at these doses of γ-ray, the radiation burn had manifested in control mice by the end of first week after the LI, and pathomorphological effect reached the maximum by the 20–25th day. At the same time, the effect of RSR in these animals clearly increased along with the dose of γ-radiation (Figure 3A–D). At a dose of 20 Gy, the RSR pattern in the control mice reflected a significant preservation and functional viability of the epidermis: partial hair removal, moderate erythema and DD were observed, which, in 40–50% of animals during the period of maximum burn development, in some places acquired the character of an islet EE. At a dose of 25 Gy, the scale of epidermal damage increased: the majority of the control mice had a widespread islet EE, which, in some mice, became confluent. Complete damage and rejection of the epidermis manifested in control mice at a dose of 30 Gy. Bright erythema, severe skin edema and widespread confluent EE, sometimes accompanied by pinpoint bleeding, developed in most animals. At a dose of 35 Gy, significant damage to the dermis and underlying tissues also appeared in control group: in 30% of animals, drainage EE was complicated by bleeding, the formation of ulcerative defects and, less often, the development of osteonecrosis followed by amputation of a part of the limb.

Compound T1023 in this study, as in previous experiments, objectively and clearly limited the severity of acute RSR in mice. At a dose of 20 Gy, 20% of the T1023-treated mice had no signs of radiation burn throughout the observation period. In most animals of this group, RSR was limited to moderate erythema and episodes of DD. At doses of 25–30 Gy, the RSR was manifested as widespread DD in most of the T1023-treated mice. Only 10–30% of these animals had a local islet EE. At the highest dose of γ-radiation (35 Gy), widespread islet EE developed in the majority of T1023-treated mice. It was sometimes aggravated by bright erythema, but did not acquire a drainage and ulcerative-necrotic character.

Statistical analysis showed that radioprotective effectiveness of T1023 in this experiment significantly depended on the level of radiation exposure (Figure 2A–D). At a low effective dose of γ-radiation (20 Gy), the protective effect of T1023 was weakly expressed and remained within the very border of significance (*p* = 0.09230; *p* = 0.06451 during the period of maximum manifestation of the burn). However, at doses of γ-radiation 25–35 Gy, the radioprotective effect of T1023 had a high level of statistical significance practically at all periods of observation.

Using these data, we have tried to quantitatively assess the radioprotective effectiveness of T1023 in relation to the skin. We used the degree of RSR on RTOG scale during the period of maximum manifestation of radiation burn as a measure of the toxic effect of γ-radiation. Calculations showed that the dose dependences of RSR in control and T1023-treated mice in the range of moderate toxic effects (RSR 1.0–3.5) are satisfactorily (*p* < 0.01) described by log-linear regressions (Figure 3E). According to these regressions, the estimates for DMF for T1023 (ratio of equal toxic doses values ± T1023) are in the range of 1.4–1.7. These estimates of DMF for acute RSR in γ-ray LI mice are close to the DMF value (1.6) for 30-day survival of total body γ-irradiated mice [18]. This testifies that T1023 hypoxic effect equally effectively increases the radioresistance of both organs critical for the development of acute radiation syndrome (ARS) and other normal somatic tissues.

### 2.2. The Radioprotective Effect of T1023 Does Not Develop in the Malignant Tissues of Solid Tumors

The modifying effects of T1023 during RT were investigated in two transplanted solid tumors of different histogenesis: M-1 sarcoma (M1S) in rats and Ehrlich solid carcinoma (ESC) in mice.

**Figure 4:**
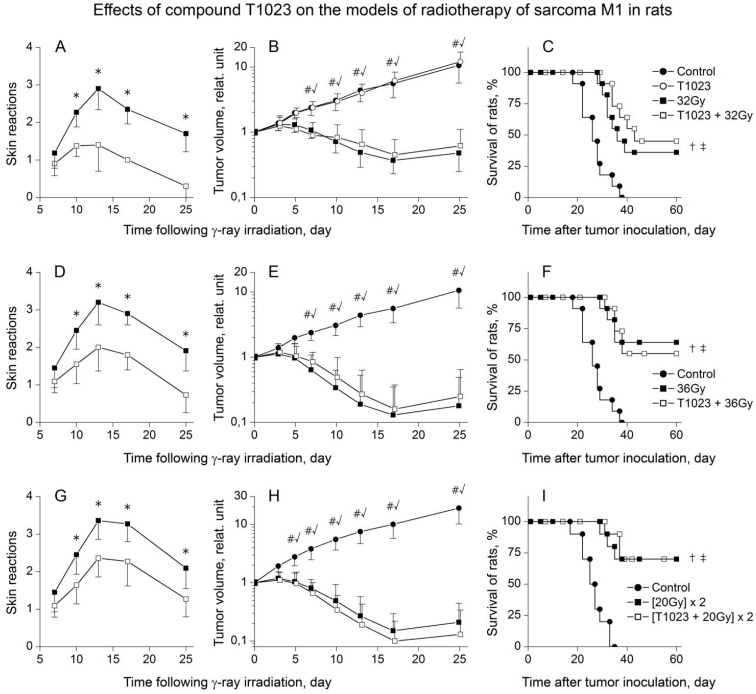
T1023 (75 mg/kg, i.p., 30 min before each LI) effects on the models of RT of M1S in rats (**A**–**C**) during single γ-ray LI at 32 Gy, (**D**–**F**) during single γ-ray LI at 36 Gy and (**G**–**I**) two fractions γ-ray LI at 20 Gy with 48 h interval. (**A**,**D**,**G**) RSR dynamics in untreated and T1023-treated rats. Assessment of the RSR degree was carried out according to the RTOG scale. Graphical deviations correspond to SD (*n* = 14–15 per point). * Significantly different RSR in ±T1023 rats ((**A**): *p* = 0.01722, *p* = 0.00293, *p* = 0.00136, *p* = 0.00087; (**D**): *p* = 0.02012, *p* = 0.00463, *p* = 0.00310, *p* = 0.00687; (**G**): *p* = 0.03062, *p* = 0.01345, *p* = 0.00952, *p* = 0.02789, respectively). (**B**,**E**,**H**) Growth curves of M1S. Tumor volume indices for each animal were normalized to the initial tumor volume on the day of the beginning of the experimental treatment. Graphical deviations correspond to SD (*n* = 14–15 per point). # Significantly different tumor volume in control vs. γ-ray-treated rats ((**B**): *p* = 0.01072, *p* = 0.00790, *p* = 0.00109, *p* = 0.00006, *p* < 0.00001; (**E**): *p* = 0.00565, *p* = 0.00224, *p* = 0.00013; *p* < 0.00001, *p* < 0.00001; (**H**): *p* = 0.01552, *p* = 0.00927, *p* = 0.00024, *p* = 0.00004, *p* < 0.00001, *p* < 0.00001, respectively). √ Significantly different tumor volume in control vs. (T1023 + γ-ray)-treated rats ((**B**): *p* = 0.00976, *p* = 0.00832, *p* = 0.00227, *p* = 0.00008, *p* < 0.00001; (**E**): *p* = 0.00739, *p* = 0.00314, *p* = 0.00023; *p* < 0.00001, *p* < 0.00001; (**H**): *p* = 0.01223, *p* = 0.00721, *p* = 0.00016, *p* = 0.00002, *p* < 0.00001, *p* < 0.00001, respectively). (**C**,**F**,**I**) Survival of rats. Survival diagrams were plotted using the Kaplan–Meier method. † Significantly different survival of control vs. γ-ray-treated rats ((**C**): *p* = 0.00881; (**F**): *p* = 0.00482; (**H**): *p* = 0.00049, respectively). ‡ Significantly different survival of control vs. (T1023 + γ-ray)-treated rats ((**C**): *p* = 0.00711; (**F**): *p* = 0.00573; (**H**): *p* = 0.00032, respectively).

In the first series of experiments, the effect of T1023 (75 mg/kg, i.p., 30 min before each radiation exposure) on acute RSR and antitumor effects was studied using models RT of M1S in rats with single γ-ray LI at doses of 32 and 35 Gy or two fractions γ-ray LI at doses 20 Gy with 48 h interval (for 14–15 rats per group). The results of this study showed that the presence of a tumor on the thigh of animals did not significantly affect the radiosensitivity of normal tissues. The development of RSR in terms of the timing and pathomorphological manifestations proceeded in these animals in approximately the same way as in healthy T1023-untreated rats. The difference was the presence of a rather pronounced permanent skin edema in the area of the tumor node, which somewhat aggravated the course of RSR, contributing to the transition of EE to the confluent form. In general, all the RT variants used caused the development of pronounced RSR in control rats by the 13–15th day after LI. Their morphological severity increased along with the total focal dose (TFD) of γ-ray (Figure 4A,D,G). Further, the processes of inflammation and regeneration of radiation damage to normal tissues of the thigh in these rats proceeded in the same way as in healthy T1023-untreated rats. Thus, the preventive use of T1023 in all RT variants realized a significant radioprotective effect on normal tissues of the irradiated thigh of tumor-bearing rats. There was a decrease in the severity of the morphological manifestations of radiation burn, and a statistically significant limitation of the RSR degree on RTOG scale in T1023-treated rats.

At the same time, in the tissues of M1S growing on the irradiated limb of rats, no radioprotective effect of T1023 was manifested in all RT variants (Figure 4B,F,H). Regardless of the use of T1023, γ-ray LI caused a long (15–17 days) growth retardation of M1S and a pronounced regression (5–10 times) of tumor nodes, which increased along with TFD of γ-ray. The growth dynamics of irradiated M1S in control and T1023-treated rats did not differ statistically in all experiments at all stages of observation. Moreover, T1023 did not weaken the overall efficacy of anticancer therapy (Figure 4C,F,I). The used RT variants significantly increased the lifespan of control and T1023-treated rats, and caused a stable remission of tumor growth in a large part of these animals (35–70%). The proportion of such ‘cured’ animals also increased along with TFD of γ-ray and did not decrease with the use of T1023.

**Figure 5:**
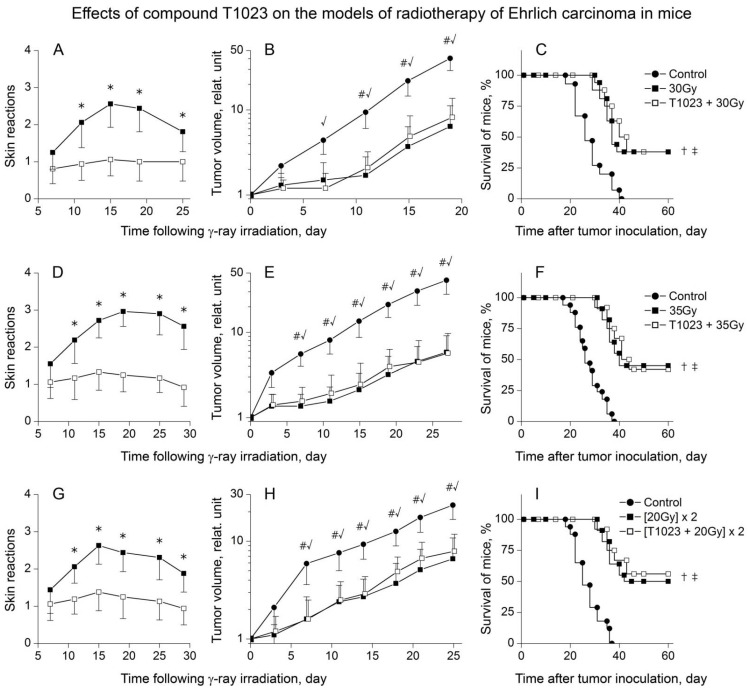
T1023 (75 mg/kg, i.p., 30 min before each LI) effects on the models of RT of ESC in mice (**A**–**C**) during single γ-ray LI at 30 Gy, (**D**–**F**) during single γ-ray LI at 35 Gy and (**G**–**I**) two fractions γ-ray LI at 20 Gy with 48 h interval. (**A**,**D**,**G**): RSR dynamics in untreated and T1023-treated mice (*n* = 20–25 per point). Explanations and designations are similar to Figure 4. * Significantly different RSR in ±T1023 mice ((**A**): *p* = 0.02889, *p* = 0.00827, *p* = 0.01194, *p* = 0.03284; (**D**): *p* = 0.03117, *p* = 0.00519, *p* = 0.00034, *p* = 0.00041, *p* = 0.00757; (**G**): *p* = 0.02530, *p* = 0.00832, *p* = 0.01159, *p* = 0.01892, *p* = 0.02275, respectively). (**B**,**E**,**H**): Growth curves of ESC (*n* = 20–25 per point). Explanations and designations are similar to Figure 4. # Significantly different tumor volume in control vs. γ-ray-treated mice ((**B**): *p* = 0.00790, *p* = 0.01011, *p* = 0.00529; (**E**): *p* = 0.00835, *p* = 0.00924, *p* = 0.00301; *p* = 0.00143, *p* = 0.00092, *p* = 0.00065; (**H**): *p* = 0.01645, *p* = 0.01393, *p* = 0.00970, *p* = 0.01148, *p* = 0.00971, *p* = 0.00642, respectively). √ Significantly different of tumor volume in control vs. (T1023 + γ-ray)-treated mice ((**B**): *p* = 0.00988, *p* = 0.00887, *p* = 0.02017, *p* = 0.00780; (**E**): *p* = 0.00983, *p* = 0.01709, *p* = 0.00566; *p* = 0.00292, *p* = 0.00128, *p* = 0.00095; (**H**): *p* = 0.01018, *p* = 0.01236, *p* = 0.00855, *p* = 0.02249, *p* = 0.02417, *p* = 0.01723, respectively). (**C**,**F**,**I**): Survival of mice. † Significantly different of survival of control vs. γ-ray-treated mice ((**C**): *p* = 0.01373; (**F**): *p* = 0.00856; (**H**): *p* = 0.00611, respectively). ‡ Significantly different of survival of control vs. (T1023 + γ-ray)-treated mice ((**C**): *p* = 0.01287; (**F**): *p* = 0.00833; (**H**): *p* = 0.00547, respectively).

In the second series of experiments, T1023’s (75 mg/kg, i.p., 30 min before each radiation exposure) influence on acute RSR was studied on the RT of ESC model in mice (single γ-ray LI at doses of 30 and 35 Gy or two fractions γ-ray LI at doses 20 Gy with 48 h interval, 20–25 mice per group). The radiation modifying effects of T1023 in this model, in general, were similar. T1023 effectively protected normal tissues of tumor-bearing mice and statistically significantly limited the severity of RSR in T1023-treated mice in all RT variants (Figure 5A,D,G). However, as in M1S, in the tissues of ESC growing on the irradiated limb of mice, the radioprotective effect of T1023 did not realize, did not modify the antitumor effects of γ-ray (Figure 5B,E,H) and did not impair the overall therapeutic efficacy of RT (Figure 5C,F,I).

**Figure 6:**
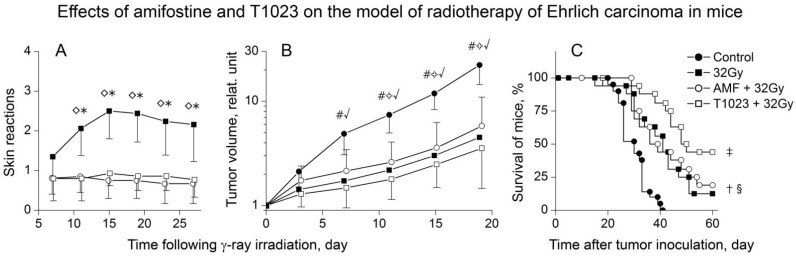
Effects of amifostine (250 mg/kg, single, i.p., 30 min before LI) and compound T1023 (75 mg/kg, single, i.p., 30 min before LI) on the model of RT of ESC in mice during single γ-ray LI at 32 Gy. (**A**) RSR dynamics in untreated, AMF-treated and T1023-treated mice (*n* = 18–20 per point). Explanations and designations are similar to Figure 4. ◊ Significantly different RSR in control vs. AMF-treated mice (*p* = 0.010192, *p* = 0.00145, *p* = 0.00562, *p* = 0.01028, *p* = 0.01405, respectively). * Significantly different RSR in control vs. T1023-treated mice (*p* = 0.00839, *p* = 0.00376, *p* = 0.00730, *p* = 0.01332, *p* = 0.01959, respectively). (**B**) Growth curves of ESC (*n* = 18–20 per point). Explanations and designations are similar to Figure 4. # Significantly different tumor volume in control vs. γ-ray-treated mice (*p* = 0.00907, *p* = 0.00471, *p* = 0.00332, *p* = 0.00299, respectively). ✧ Significantly different tumor volume in control vs. (AMF + γ-ray)-treated mice (*p* = 0.01109, *p* = 0.00941, *p* = 0.01027, respectively). √ Significantly different tumor volume in control vs. (T1023 + γ-ray)-treated mice (*p* = 0.00798, *p* = 0.00174, *p* = 0.00083, *p* = 0.00055, respectively). (**C**) survival of mice. † Significantly different survival of control vs. γ-ray-treated mice (*p* = 0.03137). § Significantly different survival of control vs. (AMF + γ-ray)-treated mice (*p* = 0.02112). ‡ Significantly different survival of control vs. (T1023 + γ-ray)-treated mice (*p* = 0.00129).

To assess the prospects of T1023 as a possible means of preventing complications of RT, we conducted a comparative study of the radiation-modifying effects of T1023 (75 mg/kg, single, i.p., 30 min before LI) and amifostine (250 mg/kg, single, i.p., 30 min before LI) using experimental RT of ESC in mice with single γ-ray LI at dose of 32 Gy. Despite the difference in the mechanisms of radioprotective activity, both these radioprotectors showed similar effects. T1023 and amifostine provided protection of the normal tissues of the irradiated tumor-bearing mice with equal effectiveness (Figure 6A). Furthermore, the radioprotective effect of both T1023 (1/4LD10) and amifostine (1/2LD10) was not manifested in ESC tissues, it did not significantly modify the antitumor effects of γ-ray (Figure 6B) and did not weaken the overall antitumor efficacy of RT (Figure 6C).

## 3. Discussion

The problem of developing pharmacological agents for the prevention and treatment of RT toxic effects have currently attracted considerable attention. The objects of research and development in this area are an extremely wide range of synthetic and biotechnological compounds with various types of biochemical and pathophysiological activity: the ability to limit the formation of primary radiation damage, modulate the processes of cell death, the activity of post-radiation repair, the course of immune-inflammatory processes and fibrogenesis [20,21,22,23].

The ability of ‘direct’ radioprotectors (that are effective during the physical and physicochemical stages of injury) to limit the development of radiation toxic effects seems to be quite natural, since primary molecular damage is the pathophysiological basis of such pathologies. This is confirmed by the presence of such abilities in aminothiol radioprotectors [24,25,26], adrenergic and serotonergic hypoxic radioprotectors [27,28,29], antioxidants [30,31] and inhibitors of radiolysis products [32,33]. The results of this study suggest that NOS inhibitors may complement the list of such agents.

The prophylactic effect of T1023 against ARS (DMF—1.6–1.9) develops according to physiological mechanisms that are characteristic, among others, for the agonists of α1B-adrenoreceptors and serotonin 5-HT2-receptors [16,18]. Rapid and pronounced vasoconstriction causes reflex changes in cardiac activity (decrease in strength and frequency of contractions) that limit systemic blood flow (for T1023: cardiac output is reduced by 40–50% within 90–120 min). The resulting transient hypoxia promotes to limit alteration under radiation exposure [17,18].

Considering such a mechanism of radioprotective activity, it is quite natural that T1023 is also capable of reducing the toxic effects of γ-radiation in normal tissues. In this work, we have shown that the prophylactic use of T1023 in the optimal radioprotective dose (75 mg/kg; 1/4 LD10) leads to effective (DMF—1.4–1.7) and pronounced limitation of the development of radiation damage to the skin and underlying tissues in mice and rats. The histological data confirmed that these effects of T1023 developed due to a decrease in radiation alteration of normal tissues and the preservation of the functional activity of cell populations that are critical in the development of radiation burn.

Means of prevention of RT complications, acceptable for clinical use, must be protective for normal tissues; it must not defend tumor cells and weaken the antitumor efficacy of radiation exposure. For example, the clinical radioprotector amifostine has such ability. The selectivity of its effects is associated with the accumulation of its active metabolite WR-1065, mainly in normal tissues, due to hypovascularization of solid tumors and low expression of alkaline phosphatase in tumor cells [24,25].

The results of our research on RT models of different solid tumors (ectodermal ESC in mice and mesodermal M1S in rats) showed that T1023 also fully implements the selective radioprotective effect. T1023 significantly limited the severity of RSR in normal animal tissues with all variants of RT (single or hypofractionated γ-ray LI). However, no manifestations of the radioprotective effect of T1023 in the malignant tissues of M1S and ESC were observed. The modifying effect of T1023 was completely absent both on the antitumor effects of γ-ray and on the RT efficacy indicators (animal survival, the proportion of animals cured). Comparison of T1023 and amifostine in the RT ESC model in mice showed that both radioprotectors in doses effective for ARS prophylaxis (T1023—75 mg/kg, 1/4 LD10; amifostine—250 mg/kg, 1/2 LD10) have similar effects. Both means implement equally effective selective protection of normal tissues. It is important to emphasize that T1023 was used in a rather less toxic dose than amifostine.

We do not yet have experimental data that directly reflect the mechanism of the selectivity of T1023 radioprotective effect, and we plan to study these issues in the near future. However, as with amifostine, the selectivity of T1023 appears to be due to the pathophysiology of solid tumors. It is known that atypical angioarchitecture and functional insufficiency of the vascular network of such neoplasias determine the presence of chronic intratumoral hypoxia in their tissues [34,35]. According to clinical studies in solid human tumors, about 34% of cells are in deep chronic hypoxia (pO_2_ < 5 mm Hg), regardless of histogenesis and stage of the process, which halves their radiosensitivity. In contrast, in normal tissues such fraction does not exceed 0.5% [36,37]. Under these conditions, T1023-induced transient hypoxia significantly alters the level of oxygenation and radiosensitivity of normal tissues. This provides prevention of ARS for γ-ray total body irradiation and toxic effects for γ-ray LI. However, in tissues of solid tumors, the scale of modification of oxygenation and radiosensitivity can be significantly limited by the initial intratumoral hypoxia and the initial radioresistance of neoplasia. In addition, the T1023 selectivity can also be facilitated by functional insufficiency of tumor vasculature, which, among other things, manifests in a weak, unstable and often paradoxical reaction of vessels and tumor blood flow to the action of vasopressors and vasodilators [38,39,40].

In general, this work was the first step in studying the possibility of using T1023 for the prevention of RT complications, and many important aspects have remained outside the scope of this study. In particular, it is necessary to study the radiation-modifying effects of T1023 on models of multifractionated RT, close to the methods used in clinical practice. The study of T1023 radioprotective effects with its locally use in the form of applications or cavity instillations is also relevant.

Another important and essentially unexplored question is its ability to resist the development of late radiation damage. So far, we have taken the first step in this direction. Prophylactic use of T1023 (75 mg/kg, single, i.p., 30 min before LI) significantly (by 40%) reduced the content of fibrous seals in the lung parenchyma and contributed to the preservation of its normal histostructure six months after thoracic γ-ray irradiation at dose of 12.5 Gy on a model of radiation fibrosis in rats [41]. These results allow us to plan detailed studies of T1023 capabilities on models of distant organ-specific radiation reactions.

## 4. Materials and Methods

### 4.1. Animals

Female of outbred ICR mice (CD-1; 4–5 months old, 27–31 g body weight) and male Wistar rats (3–4 months old, 200–270 g body weight) were used in these studies. Animals were purchased from the Biomedical Technology Scientific Center of Federal Biomedical Agency of Russia (Moscow). Animals were housed in T-3 and T-4 cages under natural light conditions with forced ventilation 16 times/h, at a room temperature 18–20 °C and relative humidity 40–70%. Animals had free access to water and rodents PK-120-1 feed (Laboratorsnab Ltd., Moscow, Russia). Animal studies were approved by the A. Tsyb Medical Radiological Research Center (MRRC) Ethical Committee and were performed in accordance with generally accepted standards for the animal treatment, based on standard operating procedures of the A. Tsyb MRRC, in accordance with the rules and requirements of the European Convention ETS/STE No. 123 and international standard GLP (OECD Guide 1:1998).

### 4.2. Drugs

Compound T1023 was synthesized in the laboratory of radiation pharmacology of the A. Tsyb MRRC. The methods used in-laboratory of the synthesis, isolation and purification of compound T1023 [18] provided a stable quality of a substance with a content of 1-isobutanoyl-2-isopropylisothiourea hydrobromide of more than 95% and a total content of related and extraneous impurities of less than 1% of dry weight. Amifostine in form of powder for preparing injection solution (ethyol; USB Pharma, Brussels, Belgium) was used as a reference drug. T1023 and amifostine were used ex tempore in the form of aseptic aqueous solutions in water for injection (JSC Dalchempharm, Khabarovsk, Russia). In all experiments, T1023 was administered as a single i.p. injection at a dose of 75 mg/kg, 30 min before irradiation (the optimal dose and time for ARS prevention [18]), and in cases of fractionated irradiation, at a dose of 75 mg/kg, 30 min before each radiation exposure. Amifostine was administered as a single i.p. injection at a dose of 250 mg/kg, 30 min before irradiation. All untreated control animals in all experiments were administered 0.9% sodium chloride for injection (JSC Dalchempharm) in equivalent volume.

### 4.3. Radiation Exposure

In all experiments, 1 day before the radiation exposure or before inoculation of experimental neoplasias, the hair was removed from all sides of the animal’s (mice and rats) right hind limb with a trimmer Moser ChroMini Type 1591 (Moser, Schwarzwald, Germany). LI with ^60^Co of the limb of healthy animals and animals with inoculated syngeneic tumor was carried out using research facility Luch-1 (Russia). Its technical characteristics and used dosimetric support are given in [18]. Four animals were placed in individual acrylite containers, from which irradiated limbs were taken out and fixed in the irradiation field (45 mm × 45 mm for mice, 75 mm × 75 mm for rats) and the animals’ bodies were covered with a lead plate 70 mm thick. Irradiations of the animals’ limbs were performed with a static position of γ-ray source in the dorsal-ventral irradiation geometry at a dose rate of 1.5–3.0 Gy/min at doses of 20–40 Gy or two fractions at doses 20 Gy with 48 h interval.

### 4.4. Tumor Models

Two experimental tumor models were used—M-1 sarcoma (M1S) in rats and Ehrlich solid carcinoma (ESC) in mice. Both neoplasias were obtained from a bank of tumor materials of N.N. Blokhin National Medical Research Centre of oncology of the Health Ministry of Russia. The strain M1S was maintained on male Wistar rats, the strain ESC on female Balb/c mice. On the 8–10th day of tumor growth, tumor nodules were isolated for preparing tumor cells suspensions in 199 medium (Paneco Ltd., Moscow, Russia) with a concentration of 107 cells/mL. M1S cell suspension was transplanted to male Wistar rats by s.c. injection of 3 × 106 tumor cells, ESC cell suspension was transplanted to female outbreed mice by s.c. injection of 2 × 106 tumor cells. Tumors were inoculated in the lateral surface of the right thigh, where the hair was previously removed.

### 4.5. Radiation-Induced Skin Reactions

The toxic effects of γ-radiation were estimated by RSR.

*Macroscopic studies* of clinical and morphological manifestations of acute radiation-induced reactions of skin and its appendages as well as the nature and dynamics of inflammatory and regenerative processes were evaluated every 3–4 days after irradiation on the irradiated limb of healthy and tumor-bearing mice and rats. The severity of RSR was assessed by the six-degree scale RTOG/EORTC-1995 [19,42]. T1023 and amifostine effects were assessed by comparison of the clinical and pathological picture and the course of acute RSR in experimental groups of animals. The statistical evaluation of intergroup differences for different observation periods was made according to the RTOG scale.

*Histological study* of T1023 effect on the RSR progression was carried out on rats on the 15th day after γ-ray LI at dose of 40 Gy. Two transverse skin fragments with underlying tissues about 2 cm long were isolated from each animal at the border of the maximally damaged and visually unchanged skin of the thigh, and they were immersed in Bouin’s fluid for 24 h and washed with 70% ethanol. The oriented tissue samples were dehydrated and embedded in Histomix^®^ (BioVitrum Ltd., St. Petersburg, Russia). For morphological studies, sections with a thickness of 5 μm, obtained with a Leica RM2235 microtome (Germany), were stained with hematoxylin and eosin (H&E; Biovitrum Ltd.). To identify actively proliferating cells and vascular structures on serial sections, immunohistochemical staining was also performed with polyclonal rabbit antibodies to the nuclear antigen of proliferating cells, PCNA (Invitrogen, PA5-27214; 1:100), and monoclonal rabbit antibodies to the endothelial marker, CD31 (Abcam, Cambridge, UK, ab182981; 1:250). To visualize these antibodies on sections, secondary goat antibodies to rabbit IgG conjugated with horseradish peroxidase (Abcam, ab205718; 1:1000), which was detected by diaminobenzidine (Liquid DAB+, Dako, K3468), were used. Cell nuclei were stained with *Mayer’s Hematoxylin*. Histological sections of tissues of the irradiated limbs of control and T1023-treated rats, stained with H&E and antibodies to PCNA and CD31, were examined under an Axio Imager A1 microscope (Zeiss AG, Jena, Germany) at three levels of magnification with microphotography on a PowerShot A640 digital camera (Canon).

### 4.6. Antitumor Effects

The irradiation of the animal limbs with grafted tumors was carried out on the 7–8th day after transplantation, when the tumor nodules in all animals had already reached reliably measurable sizes—60–90 mm^3^. The sizes of tumor nodes were measured with caliper and their volumes were estimated in the approximation *V* = *abc* × (*π*/6), where *a*, *b* and *c* are the diameters in orthogonal planes. From this moment, the volumes of tumors in animals were assessed every 2–3 days. When analyzing the data, the relative dynamics of the neoplasias growth was evaluated. For this, the indices of the tumor volume of each animal at different stages of observation were normalized to the initial tumor volume in this animal (on the 7–8th day after inoculation). The antitumor effects of γ-rays and the modifying effects of T1023 and amifostine were assessed by intergroup statistical comparison of tumor volume at different observation times, the length of the tumor growth inhibition, the survival of animals with tumor and the number of cured animals. Animals that had marked tumor regression after RT and did not have recurrent growth of neoplasia over the next 60 days were considered cured.

### 4.7. Statistical Analysis

Standard parameters of variation statistics were calculated for all experimental data and their values are given as M ± SD. For paired comparison of RSR degrees, the level of significance of differences was assessed using the Mann–Whitney U test; for multiple comparisons of RSR degrees and tumor volumes, using the Kruskal–Wallis ANOVA by ranks with post hoc Mann–Whitney U test by Bonferroni–Holm multiple test procedure; and for multiple comparisons of survival diagrams, using the χ^2^ test with post hoc Cox F test by Bonferroni–Holm procedure. Statistical significance of regressions was assessed using χ^2^ criterion. In all cases, effects, differences or dependencies were considered statistically significant at the 5% level.

## 5. Conclusions

These data allow a broader assessment of the radio-modifying capabilities of NOS inhibitors, particularly T1023. The results indicate that the vasoactive hypoxic mechanism of radioprotective activity allows T1023 to implement effective and selective prevention of acute radiation damage to normal tissues without weakening the effectiveness of radiotherapy of different solid tumors (ectodermal ESC in mice and mesodermal M1S in rats). This confirms that NOS inhibitors are of significant interest for radiation pharmacology and can create new opportunities in the development of effective and safe means of preventing complications of radiation therapy.
